# The Value of Empirical Data for Estimating the Parameters of a Sociohydrological Flood Risk Model

**DOI:** 10.1029/2018WR024128

**Published:** 2019-02-15

**Authors:** M. H. Barendrecht, A. Viglione, H. Kreibich, B. Merz, S. Vorogushyn, G. Blöschl

**Affiliations:** ^1^ Centre for Water Resource Systems Vienna University of Technology Vienna Austria; ^2^ GFZ German Research Centre for Geosciences, Section Hydrology, Telegrafenberg Potsdam Germany; ^3^ Institute of Earth and Environmental Sciences University of Potsdam Potsdam Germany

## Abstract

In this paper, empirical data are used to estimate the parameters of a sociohydrological flood risk model. The proposed model, which describes the interactions between floods, settlement density, awareness, preparedness, and flood loss, is based on the literature. Data for the case study of Dresden, Germany, over a period of 200 years, are used to estimate the model parameters through Bayesian inference. The credibility bounds of their estimates are small, even though the data are rather uncertain. A sensitivity analysis is performed to examine the value of the different data sources in estimating the model parameters. In general, the estimated parameters are less biased when using data at the end of the modeled period. Data about flood awareness are the most important to correctly estimate the parameters of this model and to correctly model the system dynamics. Using more data for other variables cannot compensate for the absence of awareness data. More generally, the absence of data mostly affects the estimation of the parameters that are directly related to the variable for which data are missing. This paper demonstrates that combining sociohydrological modeling and empirical data gives additional insights into the sociohydrological system, such as quantifying the forgetfulness of the society, which would otherwise not be easily achieved by sociohydrological models without data or by standard statistical analysis of empirical data.

## Key Points

- Socio‐hydrological flood modeling and empirical data are combined to quantitatively describe the human‐flood system of the Elbe in Dresden
- The approach presented here allows for understanding the feedbacks and dynamics of specific human‐flood systems
- With this approach it is possible to quantify parameters, like societal forgetfulness, that cannot be easily obtained with other methods

## 1 Introduction

In flood risk management the aim is to reduce the overall risk, which can be done by reducing the hazard (i.e., the frequency/magnitude of flooding), the exposure of people, and properties or their vulnerability to flooding (Kron, 2005). However, changing one of those risk components may lead to unexpected behavior of the system as a whole, resulting in phenomena like the levee effect (the increase of exposure due to the nonoccurrence of flooding) or the adaptation effect (the nonlinear relation between flood severity and loss; see, e.g., Di Baldassarre et al., 2015). In the field of sociohydrology the aim is to study phenomena that result from the long‐term interactions between society and hydrology (Blair & Buytaert, 2016; Sivapalan et al., 2012; Sivapalan & Blöschl, 2015). These human‐flood interactions have been explored in the past either by using dynamic sociohydrological models or by empirical data analyses.

### 1.1 Sociohydrological Flood Models

Di Baldassarre et al. (2013, 2015) developed conceptual models to describe the dynamics of the human system that lead to the levee and adaptation effects. They simulate the differences in behavior between technological societies dealing with flood risk by building flood protection, and green societies, which deal with flood risk by reducing the exposure in the floodplain. Viglione et al. (2014) used the same type of model to further investigate the different approaches to flood risk management and the role of different societal characteristics (collective memory, risk‐taking attitude, and trust). These studies use stylized models to describe the behavior of a hypothetical system and explore the development of the system variables by varying synthetic input data and parameter values. In a similar way, Girons Lopez et al. (2017) developed a model to study the impact of society's preparedness on flood loss mitigation by early warning systems. They assume that social preparedness can be approximated by the recency of flood experience. Yu et al. (2017) include in their model social processes such as social norms and collective actions, instead of the social memory variables used by the previous models. All these studies show the potential of using sociohydrological flood models to explain the long‐term dynamics of human‐flood systems (Barendrecht et al., 2017).

While these studies describe general phenomena in a stylized way, only a few models have been developed to describe the dynamics of a particular case study. Chen et al. (2016) use a sociohydrological model to mimic the feedbacks due to shifts in value of flood protection and wetland conservation in the Kissimmee basin, United States, and the model proposed by Yu et al. (2017) is based on the interactions observed in the coastal system of Bangladesh. Ciullo et al. (2017) use data from the human‐flood systems of Rome and Bangladesh and compare them to the outputs of the model developed by Di Baldassare et al. (2015). They show that technological societies who build protection to deal with flood risk have lower flood risk (i.e., expected loss per year) but are more prone to individual catastrophic event loss and are less resilient than green societies that deal with flood risk using nonstructural measures.

**Figure 1:**
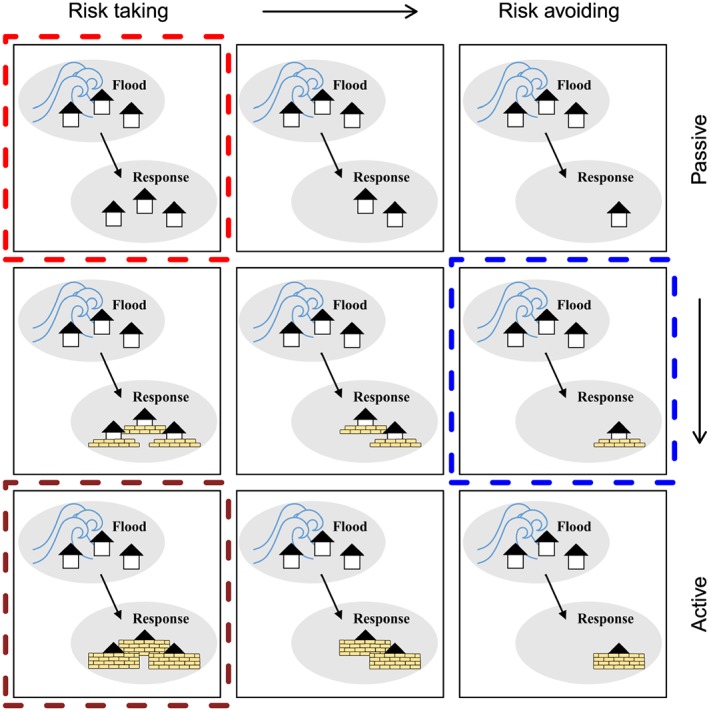
The panels present different behavioral attitudes after experiencing a flood. From left to right the panels show a change in risk taking attitude from risk taking to risk avoiding (i.e., how quickly households move away from the river after experiencing flooding). From top to bottom the panels show a change in activeness from passive to active (i.e., how quickly they implement measures for reducing loss after experiencing flooding).

Even though Chen et al. (2016), Yu et al. (2017), and Ciullo et al. (2017) have developed their models based on specific case studies, they compare their model outputs to real world data in a qualitative way, by looking at similarities between simulated dynamics and data, without quantifying the parameters of their models through a formal estimation procedure. We argue that using data to estimate the parameters of sociohydrological models may uncover unique features of the particular systems under study and allow for a comparison between them. Taking Viglione et al. (2014) as an example, determining the parameter values of the model can help explain whether the society in a specific case study is a rabbit (risk avoiding) or a lion (risk taking), whether it is an elephant (forgetting slowly) or a cicada (forgetting quickly), and how similar/different it is compared to other cases. This is illustrated in Figure 1, which shows possible behavioral attitudes in terms of risk taking attitude (i.e., how quickly households move away after experiencing flooding) from left to right and in terms of how active households are (i.e., how many measures they take to reduce loss after experiencing flooding) from top to bottom. If, for example, in a certain case study, the parameters of a model that includes these dynamics are estimated, this particular society can then be placed in the top left panel indicated by the red dashed square. This then could be used to determine whether it might be worth implementing policies that move this society to the panel marked in blue (middle right). Defining the range of different behavioral attitudes also allows for comparison of the society circled in red with another society that is located in the panel marked in brown (bottom left), and to investigate why these societies are behaving differently. Moreover, using data to estimate the parameters of a dynamic model not only provides quantitative information about the parameter values but also is a test of whether the hypothesis about the real world processes that generate the observed behavior (i.e., the hypothesis that the model structure represents the real world) is consistent with the observed data (Oliva, 2003). This does not mean that one model can be verified or validated as the true representation of reality. As explained by Oreskes et al. (1994) this cannot be fully achieved for open systems like the ones in sociohydrology. This means that the model/hypothesis can be compared to, updated, or substituted by other models/hypotheses as discussed by Troy et al. (2015).

### 1.2 Empirical Studies

Several attempts have been made to find factors that influence the relationship between damage and flood magnitude, using empirical research methods. The relationships between flood risk perception, awareness, protective behavior (e.g., the uptake of precautionary measures), and flood damages have been studied using interview data collected in the Czech Republic (Duží et al., 2017), Greece (Fuchs et al., 2017), Scotland (Owusu et al., 2015), Spain (Raaijmakers et al., 2008), France (Poussin et al., 2014; Poussin et al., 2015), Germany (Bubeck et al., 2012, 2013; Kreibich et al., 2005; Kreibich & Thieken, 2009; Kreibich et al., 2011; Osberghaus, 2015; Thieken et al., 2007), the Netherlands (Botzen et al., 2009), Iceland (Pagneux et al., 2011), and Italy (Miceli et al., 2008; Scolobig et al., 2012). Although some of these studies (Duzi et al., 2017; Fuchs et al., 2017) do not find correlation between flood experience and the uptake of precautionary measures, the majority of them have shown that a correlation exists and is indeed significant (Bradford et al., 2012; Bubeck et al., 2013; Kreibich & Thieken, 2009; Osberghaus, 2015; Owusu et al., 2015; Poussin et al., 2014; Wachinger et al., 2013). Aside from the direct experience of damages, other factors have been found that may influence protective behavior, like coping appraisal (Bubeck et al., 2012, 2013), maladaptive coping responses (Bubeck et al., 2013; Grothmann & Reusswig, 2006), worry (Miceli et al., 2008; Raaijmakers et al., 2008), or trust in structural protection (Scolobig et al., 2012; Wachinger et al., 2013).

Empirical studies also show that the implementation of precautionary measures can greatly reduce damages due to a flood event (Bubeck et al., 2012; Hudson et al., 2014; Kreibich et al., 2005; Poussin et al., 2015; Wind et al., 1999). How big this reduction is depends on the region and its flooding characteristics (Poussin et al., 2015), whether damage is to buildings or contents (Kreibich et al., 2005) and on the type of measure that has been implemented (Hudson et al., 2014; Kreibich et al., 2005).

While empirical research quantifies, for specific case studies, the relationships between damages and damage reducing measures, the use of empirical data also has its limitations. Most of the empirical data are collected after one specific flood event. Longitudinal studies, which undertook a survey before an event as well as repeatedly after it, are scarce, which hampers the analysis of temporal changes in awareness and precautionary behavior and their relation to flood experience (Weinstein, 1989; Osberghaus, 2017). Similarly, not many cross‐sectional data sets covering the same area at different points in time are available to unravel the temporal changes in the relationship between flood magnitude, exposure, private, and institutional risk mitigation and damages (exceptions are, e.g., Kienzler et al., 2015; Kreibich et al., 2017). Statistical analyses of empirical data are useful for finding correlations between the different variables, but they do not allow for the explicit investigation of the dynamic relationships with feedback mechanisms between the variables. Empirical studies are always limited to specific case studies, which all have their own characteristics, making it a challenge to retrieve generic, transferable results (Schröter et al., 2014). Also, risk mitigation and behavior influencing flood impacts are strongly context and location specific (Dzialek et al., 2016). For instance, socioeconomic, cultural, and environmental settings influence what options for reducing vulnerability are realistic (Kreibich et al., 2017). Approaches to tackle this challenge to retrieve generic results are comparative analyses of large sets of case studies (e.g., Blöschl et al., 2013; Kreibich et al., 2017) and theory or model driven empirical analyses (e.g., Bubeck et al., 2013; Kreibich et al., 2011). Additionally, there is the inherent difficulty of measuring awareness and human and societal level behavior (Aerts et al., 2018). Common approaches are surveys or detailed interviews with various stakeholders (e.g., Bubeck et al., 2013; Grothmann & Reusswig, 2006; Kreibich & Thieken, 2009), but uncertainty remains as to whether people act as they state. Also, time series analyses of proxies or indicators are sometimes used to investigate temporal changes in awareness and protective behavior, such as the change in flood insurance coverage, news media coverage, or number of Google searches (e.g., Hanak, 2011; Quesnel & Ajami, 2017).

### 1.3 Estimating Parameter Values of a Sociohydrological Model Using Empirical Data

The aim of this paper is to demonstrate the feasibility of estimating the parameters of a sociohydrological model using empirical data and to explore the value of data availability (i.e., amount, type, and timing of observations) for simulating the system as accurately as possible. We suggest that since both dynamic sociohydrological modeling studies and empirical data analyses have advantages and limitations, combining the two methods may exploit the positive aspects of both methods and compensate for the respective limitations.

The gain of using a sociohydrological modeling approach in combination with empirical data is that it allows for a consistent interpretation of all available data together, including their interactions. By estimating the parameters of the model, we formulate a quantified hypothesis of the real world processes that lead to the observed system behavior. Parameter estimation is more difficult for sociohydrological models than it is for regular hydrological models (Sivapalan & Blöschl, 2015) because the processes are less well known and more nonlinear, and the data are of different types (qualitative versus quantitative, more or less uncertain, etc.) and incomplete, or not complete to the same extent (like precipitation and discharge time series usually are).

In the field of system dynamics several methods have been proposed for the quantification of parameter values of nonlinear dynamic models. Parra et al. (2018) test the performance of four commonly used optimization methods that find a parameter set based on an objective function (Genetic Algorithms, Simulated Annealing, Powell's Algorithm, and Hybrid Algorithms). These methods depend on the availability of a long time series of observations and are therefore not suitable for use in sociohydrology where data availability is limited. Hosseinichimeh et al. (2016) propose a method for the estimation of parameters that does not depend on the availability of long time series of data. They use indirect inference, which is based on matching the *auxiliary statistics* (like the mean or variance of the data at one time point) of empirical and simulated data. While this method does not depend on the availability of long time series it does require a large amount of data at one point in time to be able to calculate the auxiliary statistics. Since this type of data is also not available in sociohydrology (e.g., we usually have one value for the flood loss in a certain year), indirect inference may not be feasible for application in this study.

Here we use Bayesian inference (Gelman et al., 2014) for the parameter estimation of a sociohydrological model. This method is suitable for the incorporation of the different types of data that are available in sociohydrology and allows for the use of prior information (e.g., expert knowledge) to constrain model parameters. Another advantage is that Bayesian inference does not produce point estimates for parameter values, but distributions that account for uncertainties in the parameter estimates.

In the following sections, after proposing a sociohydrological model that describes the interactions between floods, settlement density, damages, awareness, and preparedness, Bayesian inference is used to estimate the parameters of this model with empirical data for the case study of Dresden, Germany. A sensitivity analysis is performed to explore which type of data is most important and at what point in time data should be collected.

## 2 Methods

### 2.1 The Model

Risk is often defined as hazard x exposure x vulnerability (Kron, 2005). In this paper, we are considering households or residential buildings at risk of flooding. The adaptation of households to flooding is an important phenomenon that has been described extensively for the case of Germany and Dresden by Kreibich and Thieken (2009) and others (Kreibich et al., 2005; Thieken et al., 2007, 2016). Therefore, the data availability for the human‐flood system that describes the adaptation of households for the case study of Dresden is relatively high, which fits the purpose of this paper.

Hazard in the definition of risk is determined by high discharges or high water levels and their frequency of occurrence. The frequency can be reduced by increasing the protection level, through, for example, raising dike heights. Since the model aims to explain the behavior of households, while increases in structural protection level are a decision that is made by the government and institutions, the protection level is modeled as exogenous to the system, differently from Di Baldassarre et al. (2013) and Viglione et al. (2014). The protection level is assumed to be given and households' behavior to reduce the residual risk is modeled. The residual risk can be reduced by changing the exposure and/or vulnerability. Exposure is defined as the amount of residential buildings that are at risk of being flooded when high discharges occur, that is, the settlement density. Vulnerability can be reduced by increasing households' preparedness, through the implementation of precautionary measures (Kreibich & Thieken, 2009).

The following variables are defined to describe this system and its feedbacks (see also Table 1): floods (*W*), protection level (*H*), loss (*L*), settlement density (*D*), relative loss (*R*), awareness (*A*), and preparedness (*P*). Based on expert judgment and a review of the literature, these are the variables that best describe the dynamics we are interested in. Floods (*W*) and protection level (*H*) are exogenous variables. We assume the dynamics of the other variables over time can be described by the system of differential equations in equations (1a)–(1e). The system consists of only three differential equations (they have been split up into five equations for readability), which means the number of equations is small and the model is relatively simple compared to other sociohydrological studies.

(1a) $$L=\mathrm{RD}\;\left[\text{€}/\text{€}\right]$$

(1b) R=Rmax−βRexp−αRPmax−PWWmax,W>H0,W≤H€m2/€m2

(1c) $$\begin{array}{cc} \frac{dD}{dt}=U\left(1-\alpha_{D}A\right)D\left(1-\frac{D}{D_{\max}}\right) & \left[m^{2}/m^{2}\right] \end{array}$$

(1d) $$\begin{array}{cc} \frac{dA}{dt}=\alpha_{A}L\left(1-\frac{A}{A_{\max}}\right)-\mu_{A}A & \left[n_{h}/n_{h}\right] \end{array}$$

(1e) dPdt=αPdAdt1−PPmax−μPP,R>0−μPP,R=0nm/nm

**Table 1:** Model Variables and Model Parameters, n_m_ Is the Number of Precautionary Measures, n_h_ Is the Number of Households

External variable	Internal variable	Parameter	Definition	Unit
*W*			Maximum annual discharge	((m^3^/s)/(m^3^/s))
*H*			Protection level	((m^3^/s)/(m^3^/s))
*U*			Urbanization rate	(1/t)
	*A*		Awareness	(n_h_/n_h_)
	*P*		Preparedness	(n_m_/n_m_)
	*D*		Settlement density	(m^2^/m^2^)
	*R*		Relative loss	((€/m^2^)/(€/m^2^))
	*L*		Loss	(€/€)
		*W*_max_	Maximum flood discharge	(m^3^/m^3^)
		*α*_*D*_	Risk taking attitude	(1/(n_h_/n_h_))
		*D*_max_	Maximum settlement density	(m^2^/m^2^)
		*α*_*A*_	Anxiousness	(1/(€/€))
		*A*_max_	Maximum awareness	(n_h_/n_h_)
		*μ*_*A*_	Forgetfulness	(1/t)
		*μ*_*P*_	Decay rate of precautionary measures	(1/t)
		*P*_max_	Maximum preparedness	(n_m_/n_m_)
		*α*_*P*_	Activeness	((n_m_/n_m_)/(n_h_/n_h_))
		*R*_max_	Maximum relative loss	((€/m^2^/(€/m^2^))
		*β*_*R*_	Discharge to loss relationship	((€/m^2^)/(€/m^2^))
		*α*_*R*_	Effectiveness of preparedness	(1/(n_m_/n_m_))

**Table 2:** Summary of the Data

Variable	Time points	Definition	Data used	Source
*W*	1798–2013	Annual maximum discharge divided by the discharge with a 500‐year return period	Discharge time series from the gauge at Dresden	BMBF‐Project (2007) (maximum discharge from Umweltamt Dresden, personal communication, 19 July 2017)
*H*	1798–1811, 1812–1830, 1831–2010, 2011–2013	Protection design discharge divided by the discharge with a 500‐year return period	Reconstructed based on historical reports of floods	Pohl (2004), Weikinn (2000), Weikinn and Börngen (2002), Kreibich and Thieken (2009), and Landestalsperrenverwaltung des Freistaates Sachsen (2013)
*U*	1798–1879 1880–1889,	Growth rate of the settlement density	Based on growth rate of urban areas in the cities of Germany	Hanze data set (Paprotny, 2017)
	1890–1899, 1900–1909, 1910–1919, 1920–1929			
	1930–1939 1940–1949			
	1950–1959, 1960–1969,			
	1970–1974, 1975–1979, 1980–1984, 1985–1989			
	1990–1994, 1995–1999,			
	2000–2004, 2005–2009,			
	2010–2014			
*A*	2002, 2003, 2007, 2014	Percentage of households aware of flood risk	Based on survey data	Kreibich et al. (2005), Kreibich and Thieken (2009), and Thieken et al. (2016)
*P*	1798/1845, 2002, 2003, 2006, 2007, 2013, 2014	Percentage of precautionary measures taken	Based on survey data	Kreibich et al. (2005), Kreibich and Thieken (2009), and Thieken et al. (2016)
			Increase in *P* at the beginning of the period based on qualitative narrative	Poliwoda (2007)
*D*	1880, 1900, 1940, 1953, 1968, 1986, 1998	Percentage of the floodplain that is classified as urban area	Urban area based on reconstructed land use maps	Gruner (2012; floodplain maximum discharge from Umweltamt Dresden, personal communication, 19 July 2017)
			Total area based on the floodplain corresponding to maximum discharge	
*L*	1799, 2002, 2006, 2013	Residential loss after event divided by the total value of residential properties in the floodplain	Loss values based on reported values in the literature	Korndörfer et al. (2006), Kreibich et al. (2011), LfULG (2015), and Internationale Kommission zum Schutz der Elbe (2007)
			Total values based on Kleist et al. (2006)	Kleist et al. (2006)
			Value in 1799 based on historical reports and converted to Euro	Weikinn (2000), Poliwoda (2007), and Deutsche Bundesbank (2017)

Some of the equations are based on the equations formulated in Di Baldassarre et al. (2013) and Viglione et al. (2014; parts of equations (1b), (1d), and (1e)). The other equations are based on discussion about the dynamics of the system with flood risk experts (the authors of this paper, who work on flood risk quantification at the German Research Centre for Geosciences). The equations are formulated to reproduce the dynamics as expected by these flood risk experts, which we describe as follows: flooding occurs when the discharge (*W*) is higher than the design discharge of the flood protection (*H*). This leads to potential or relative loss (*R*) as described in equation (1b), that is, when W is smaller than H the relative loss is zero and as W increases the relative loss gets closer to the maximum. The actual loss (*L*) that is caused by the flooding event (equation (1a)) is equal to the relative loss (*R*) times the settlement density (*D*), since if the floodplain is empty, no loss can occur. The loss is defined as the percentage of the value in the floodplain that is lost after an event. It is thus the actual loss divided by the value of the residential property in the floodplain. In this way the effects and changes of the value of the development in the floodplain are taken into account.

The relative loss (*R*) may be lower if preparedness is higher, that is, if people take precautionary measures. According to the literature, the uptake of precautionary measures is influenced by many factors. Households have to be aware of the risk and have a high threat appraisal (Bubeck et al., 2012). Here it is assumed that households' awareness of the flood risk is mainly influenced by prior experience of flood damage. The awareness (*A*) increases with a shock when households experience loss and decreases exponentially over time when no flooding occurs (equation (1d)). How much awareness increases due to the experience of loss depends on the parameter *α*_*A*_, the *anxiousness* of the society (i.e., if the society is very anxious loss will have more effect on their awareness). Awareness decreases over time with a rate *μ*_*A*_, which is called here the *forgetfulness* of society.

Protective behavior or the uptake of precautionary measures is represented by preparedness (*P*). An increase in awareness leads to (part of) the households taking precautionary measures and increasing preparedness (equation (1e)). The fact that not all households that are aware of the risk or have a high threat appraisal take precautionary measures is represented by the parameter *α*_*P*_, the *activeness*, which determines the level of increase in preparedness due to an increase in awareness. The increase in preparedness also depends on the level of preparedness itself, that is, if preparedness is high already, the increase is less, since there is less possibility to take additional measures. Like awareness, preparedness only increases with a shock when a loss occurs, but the size of the increase does not depend on the size of the loss (it depends on the size of the awareness), this is represented with a step function (equation (1e)). Since it is assumed that when people forget about the flood risk, they will also forget how to implement or to maintain measures, the preparedness decays exponentially over time with a rate *μ*_*P*_, the *decay rate of precautionary measures*.

The literature suggests that taking precautionary measures can greatly reduce the damages caused by a flood (Bubeck et al., 2012; Kreibich et al., 2005, 2014; Poussin et al., 2015). In this model, the amount of precautionary measures that have been taken by the households in the study area is represented by the preparedness. Thus, the preparedness influences the loss (*L*) that occurs because of flooding by reducing the relative loss (*R*; equation (1b)). For the same flood magnitude a higher preparedness ensures that the relative loss is lower. How big this influence is depends on the parameter *α*_*R*_, the *effectiveness of preparedness*.

The awareness also influences the growth rate of the settlement density (*D*; equation (1c)), that is, if people experience loss because of a flood event, they may decide to slow down the development of the area or designate certain areas as part of the river (Umweltbundesamt‐Federal Environmental Agency, 2003). The parameter *α*_*D*_, the *risk taking attitude*, controls how much influence the awareness has on the growth rate. If the awareness does not have any influence, the settlement density will grow with an exogenous urbanization rate (*U*) depending on the current value of the settlement density, (i.e., if *D* is close to one, *D* will grow slowly, and if *D* is close to zero, *D* will grow faster). Other factors that may influence the decision to move into or away from the floodplain are taken into account through the parameter *α*_*D*_. Its magnitude indicates whether the growth is more influenced by flood loss or by other factors (like real estate prices and government compensation). If government compensation is high, for example, then households will have less incentive to move, even though their awareness is high. In this case *α*_*D*_ would have a low value. If households have to pay for the damage themselves and are not compensated for their loss by someone else (e.g., the government or insurance companies), *α*_*D*_ would have a high value. The settlement density influences the loss that occurs due to a flood event, since the loss will be higher if there are more households that can potentially experience damage during flooding.

All variables are defined in such a way that they are scaled between zero and one. This means that the values *R*_max_, *W*_max_, *D*_max_, *A*_max_, and *P*_max_ (equations (1a)–(1e)) are equal to one in this application of the model.

### 2.2 Bayesian Inference

The model has 12 parameters that determine its behavior over time. Table 1 shows the parameters, their descriptions, and their units. The aim is to estimate the values of these parameters from data for the human‐flood system of Dresden, thus quantifying its societal/cultural characteristics, such as *activeness* and *forgetfulness*, and determining how this particular system has developed over time.

Data come from very different sources and are of different types: qualitative versus quantitative, less uncertain versus more uncertain, many data points versus only a few data points (see section 3). Bayesian methods (Gelman et al., 2014) allow for the inclusion of all of these different types of data. Using the Bayes' theorem (equation (2)) for inference, the posterior distribution of a set of parameters is derived from a combination of observations and prior knowledge about the parameter distribution.

(2) $$p\left(\theta |y\right)=\frac{p\left(y|\theta \right)p\left(\theta \right)}{\int p\left(y|\theta \right)p\left(\theta \right)d\theta }$$

*p*(*θ*|*y*) is the posterior estimate of the distribution of the parameters given the prior information and the observations *y*. *p*(*y*|*θ*) is the likelihood of the observations, given the model parameter values, which incorporates the information about the observed values *y* and the uncertainty of these observations. *p*(*θ*) is a distribution that quantifies the prior information on the parameter values. Prior information can be implemented quite easily using more or less detail depending on the information that is available. If no information is available at all, an uninformative prior is used. Observed values can be included as data points with a specified uncertainty. As using Bayesian inference to estimate parameter values yields parameter distributions, rather than one single set of parameter values, this method naturally yields an estimation of their uncertainty (or their credibility) under the assumption that the model structure is correct.

Since the integral in the denominator of Bayes' rule is in this case not explicitly solvable, the posterior distribution of parameters is approximated using a Markov Chain Monte Carlo (MCMC) simulation method. MCMC methods are a class of algorithms for sampling from probability distributions based on constructing a Markov chain that has the desired distribution (in this case, the posterior probability model) as its equilibrium distribution (see, e.g., Gelman et al., 2014; Robert & Casella, 2004). The states of the chain after a large number of steps are then used as a sample from the desired distribution (the quality of the sample improves as a function of the number of steps). Here the software Stan (Carpenter et al., 2017) is used to perform the MCMC inference. Stan makes use of Hamiltonian Monte Carlo sampling, which speeds up convergence and parameter exploration by using the gradient of the log posterior (Stan Development Team, 2017). To solve the system of differential equations in Stan, the equations are written in finite difference form using the forward Euler method (see Text S1 in the supporting information for a more detailed description of the implementation of the Bayesian inference).

We have scaled the variables of the model between 0 and 1 by dividing all values by their maximum values, whereby the maximum values correspond to the total volume of the system under consideration (i.e., the maximum discharge is equal to the maximum discharge considered in the flood maps of the Umweltamt Dresden [personal communication, 19 July 2017], the maximum settled area is assumed equal to the system area, the maximum awareness is assumed equal to all households within the system boundaries being aware, etc.). This means that the maximum values of the variables in equations (1a)–(1e) are equal to 1. This leaves seven parameters for which a value has to be determined through the inference. The parameters *β*_*R*_ (discharge to loss relationship) and *α*_*R*_ (effectiveness of preparedness) have a similar influence on *R* (a big *β*_*R*_ and a small *α*_*R*_, has the same effect as a small *β*_*R*_ and a big *α*_*R*_). Since it is not possible to determine a value for both of them (Gelman et al., 2014) from the available data (see section 3), we have set *β*_*R*_ equal to one. The values of *α*_*R*_, *α*_*D*_, *α*_*A*_, *μ*_*A*_, *α*_*P*_, and *μ*_*P*_ are determined using Bayesian inference.

Because the evolution of some of the variables in the model is described by differential equations, the initial values of those variables have to be imposed. Since there is no data available for the initial values of the awareness (*A*_0_), preparedness (*P*_0_), and settlement density (*D*_0_), they are estimated as well.

### 2.3 Sensitivity Analysis

To explore the importance of data, and the importance of the time of measurement of the data, a sensitivity analysis is conducted. The parameters are estimated using varying combinations of timing and availability of data. The average values of the estimates for the parameter values for the case study of Dresden are used to simulate time series for all variables with the model. Then two flooding events are sampled from these time series. For each event the loss and settlement density before and the preparedness and awareness before and after the event are taken as sample observations. The timing of these samples is varied as follows: (1) two samples at the beginning of the time series (BB); (2) two samples at the end of the time series (EE); (3) one sample at the beginning and one at the end of the time series (BE); (4) two samples in the middle of the time series (MM); (5) one sample at the beginning and one in the middle of the time series (BM); (6) one sample in the middle and one at the end of the time series (ME).

The importance of the availability of the data is investigated by varying the combination of the data as follows: (1) all data: two samples for settlement density, loss, awareness, and preparedness; (2) no awareness data: two samples for settlement density, loss, and preparedness; (3) no preparedness data: two samples for settlement density, loss, and awareness; (4) no settlement density data: two samples for loss, awareness, and preparedness; (5) no loss data: two samples for settlement density, awareness, and preparedness.In the sensitivity analysis the uncertainties in the data points are all set using a variance of 0.001 times the mean and uninformative priors are used for all parameters (including the initial values; see sections 3.2 and 3.3 for an explanation of data uncertainty and prior distributions).

The bias (equation (3)) and uncertainty (equation (4)) in estimating the parameters are calculated for each combination of sample timing and included variables.

(3) $$\text{Bias}=\frac{\text{mean}\left(\hat{\theta }\right)-\theta }{\theta }$$

(4) $$\text{Uncertainty}=\frac{\mathrm{sd}\left(\hat{\theta }\right)}{\text{mean}\left(\hat{\theta }\right)}$$

where *θ* is the real parameter value and $\hat{\theta }$ the estimated one.

## 3 The Case Study

**Figure 2:**
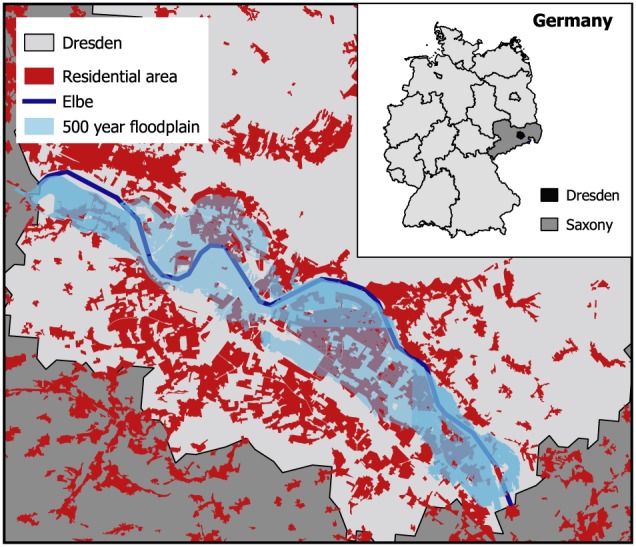
Floodplain in Dresden in Saxony, Germany, with the residential areas, the Elbe and the outlines of the floodplain for a flood with a return period of 1 in 500 years. (data sources: Administrative boundaries from https://gadm.org/index.html [accessed 1 July 2017]; river outline from http://www.diva‐gis.org/gdata [accessed 18 June 2018]; flood outline from Umweltamt Dresden, personal communication, 19 July 2017; urban area from Gruner, 2012)

The case study for which we estimate the model parameters is the city of Dresden, Germany, which is located along the Elbe River (Figure 2). Dresden experienced a long period without flooding followed by a severe flood in 2002. The flood in 2002 caused high loss and was followed by a smaller flood in 2006 and another severe flood in 2013. The damage due to the flooding in 2013 was much lower than in 2002, and it is believed that this is caused by an increase in awareness and an increased uptake of precautionary measures, such as adapted interior fitting or the use of water barriers (Thieken et al., 2016). Discharge data, damage data, and interview data that have been collected in the city of Dresden as well as qualitative information from narratives are used to estimate the parameter values of the model described in section 2.1 through Bayesian inference (section 2.2).

### 3.1 Data

**Figure 3:**
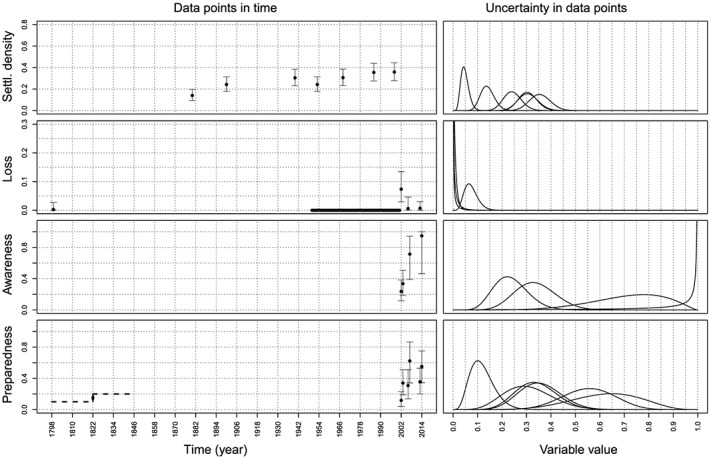
Empirical data. Figure to the left shows the values of the data in time, with the 95% uncertainty bounds. The figure on the right shows the density of each of the data points. The line of values that are zero in the loss subplot represents the knowledge that since the 1940s there have not been any flood events causing damages in Dresden. The dashed lines in the preparedness subplot represent the knowledge that the preparedness in 1845 was higher than at the beginning of the time series.

Figure 3 shows the available empirical data projected in the space of the model variables. The system considered here is spatially bounded by the outline of the maximum floodplain. In the case of Dresden the maximum floodplain corresponds to the area that would be flooded when a flood with a return period of 500 years occurs (i.e., a flood with a discharge of 6255 m^3^/s, Umweltamt Dresden, personal communication, 19 July 2017).

In the model, floods (*W*) are represented by discharges, which are scaled between zero and one by dividing them through the discharge of 6,255 m^3^/s, which would cause the maximum floodplain inundation. The unit is thus (m^3^/s)/(m^3^/s). Discharges measured at the Elbe gauge in Dresden in the period from 1798 to 2013 are used as an input (BMBF‐Projekt, 2007).

The protection level (*H*) is equal to the discharge the flood protection is designed to withstand. It is also scaled by dividing through the maximum discharge and has a unit of (m^3^/s)/(m^3^/s). The development of the protection level over the years was reconstructed based on historical reports of flooding (Pohl, 2004; Weikinn, 2000; Weikinn & Börngen, 2002). Weikinn collected and reported all historical mentions of hydrological events from CE zero until 1850. It is assumed that when a flooding is mentioned in a report, the protection level must have been lower than the discharge at that time. In addition, Kreibich and Thieken (2009) report that the last time damage occurred before 2002 was in the 1940s, which means that we assume the protection level since those years was higher than the observed discharges. Since 2011, protection levels have been increased to protect against a discharge with a return period of 100 years (Landestalsperrenverwaltung des Freistaates Sachsen, 2013).

The loss (*L*) is equal to the total residential building loss after a flood event divided by the total residential building value and has a unit of €/€. Estimated residential building loss for 2002 (average of two reported values, Korndörfer et al., 2006; Kreibich et al., 2011) and 2013 (LfULG, 2015) is divided by the residential building value as calculated by Kleist et al. (2006) for 2002 and an updated value for 2013 (and 2006) using the method described by Kleist et al. (2006). The values by Kleist et al. (2006) are for the whole city of Dresden, while the system boundary is the 500‐year floodplain. Therefore, they are first multiplied by the area of the floodplain divided by the area of the city of Dresden. For 2006, only estimated total loss is available (Internationale Kommission zum Schutz der Elbe, 2007); therefore, the residential building loss is approximated by multiplying it with the average ratio of the residential building loss and total loss for 2002 and 2013 (Kreibich et al., 2005; Landesamt für Umwelt, Landwirtschaft und Geologie, 2015).

According to Weikinn (2000) and Poliwoda (2007), the loss in 1799 was higher than the loss in 1784, which was 33556 Thaler, the loss in Thaler is transformed to Euro using estimates of the purchasing power from the Deutsche Bundesbank (Deutsche Bundesbank). Since no data is available for the total value of residential buildings in 1799, the ratio of the settlement densities in 1780 and 1998 (the years closest to 1799 and 2002 for which settlement density data are available) is assumed to be equal to the ratio of the residential building value in 1799 and 2002.

The settlement density (*D*) is the area within the system boundaries that is covered with residential buildings divided by the total area within the system boundaries and therefore has a unit of m^2^/m^2^. The settlement density within the floodplain area is estimated for seven points in time (1880, 1900, 1940, 1953, 1968, 1986, and 1998), based on the area that is reported as *residential fabric* in reconstructed land use maps (Gruner, 2012) divided by the total area of the floodplain. The settlement density grows with an urbanization rate *U*. This *U* is equal to the average growth rate of the cities in Germany and is calculated based on the HANZE land use maps (Paprotny et al., 2018).

Awareness (*A*) is defined as the number of households within the system boundaries that are aware of the flood risk divided by the total number of households within the system boundaries and has a unit of number of households/number of households. After the 2002, 2006, and 2013 flood events a telephone survey was conducted in the Elbe region with 300, 21, and 117 households, respectively (the details of the survey can be found in Kreibich et al., 2005; Kreibich & Thieken, 2009; Thieken et al., 2016). During these interviews the person in each household with the best knowledge about the flood event and the incurred loss was asked (among other questions) whether he or she experienced a flood before this one, whether they knew that the household was situated in an area at risk of flooding, and how likely he/she thought it is that they will experience damage due to a flood again. The first two questions could be answered with a *yes* or a *no*. To the last question, respondents answered on a scale from 1, very unlikely, to 6, very likely. The percentage of people that answered yes to the first or the second question in 2002 is used as an approximation of the awareness before the 2002 flood. The percentage of people that answered four, five, or six to the third question in 2002, 2006, and 2013 is used as an approximation of the awareness of the society after the 2002, 2006, and 2013 flood events, respectively. Values based on the first two questions to approximate the awareness before the 2006 and 2013 floods are not used because the percentage of people who thought that it was likely that they would experience damage due to a flood again after 2002/2006 was lower than the percentage of people that was aware of living in a floodplain before the flood of 2006/2013. Therefore, it seems that the knowledge of living in an area that is at risk of flooding is not a good approximation for the awareness before the flood events of 2006 and 2013. Note that these data are obtained from stated answers and not observed behavior. The actual behavior of people may differ from their answers to the questions asked during a survey. However, data collected through surveys are the best data available for this case study and we assume they lead to a sufficiently close approximation of the awareness.

Similarly to awareness, preparedness (*P*) is defined as the number of precautionary measures taken by the households within the system boundaries divided by the total number of precautionary measures that could have been taken by the households. Preparedness has a unit of number of measures/number of measures. The same set of interview data that is used to calculate the awareness is used to approximate the preparedness. Households were asked when they had implemented each measure of a set of precautionary measures. The available answers were *before the flood*, *during*/*after the flood*, *within the next six months*, and *not planned*. The average preparedness of society before the events in 2002, 2006, and 2013 is approximated by the total amount of measures taken by the interviewed households before the event divided by the total amount of measures that is available. The average preparedness of society after the events in 2002, 2006, and 2013 is approximated by the total amount of measures taken by the interviewed households before the event, during/after the flood event, and within 6 months after the flood event, divided by the total amount of measures that is available. In addition to the interview data, Poliwoda's (2007) description of how the society in Dresden adapted to the floods in the period between 1784 and 1845 is used as an approximation for the preparedness in that period. From this qualitative story we can deduce that the preparedness must have been higher in 1845 than in 1798.

### 3.2 Uncertainties in the Data

Since the variables are scaled between zero and one, the data and variables are assumed to be distributed according to beta distributions (whose product forms the likelihood function in equation (2)). The beta distribution can be parameterized using mean (*μ*) and a parameter *ν*, which represents the uncertainty in the data. The parameter *ν* can be expressed as

(5) $$\frac{\mu \left(1-\mu \right)}{\text{variance}-1}$$

Therefore, we quantify the uncertainties of the data in terms of their variance. The uncertainties are based on expert judgment of uncertainty across variables. We use large uncertainties, which is equivalent to saying that, for example, the awareness after the 2006 event was most probably higher than after the 2002 event. A sensitivity analysis (not shown here) revealed that the influence of the choice of data uncertainties on the results of the inference does not affect the results in a significant way.

It is assumed that the data for the settlement density are the most certain and the variance is set to be 0.005 times the mean, corresponding to a coefficient of variation ranging from 0.12 to 0.18. The uncertainty in the measurements of the loss is higher than the uncertainty in the settlement density, so for 2002 and 2013 the variance is set to 0.01 times the mean, corresponding to a coefficient of variation of 0.37 and 1.2, respectively. For the 2006 loss more assumptions are made and therefore the variance is higher than for the loss in the other years and equal to 0.03 times the mean, corresponding to a coefficient of variation of 2.24. The same holds for the loss in 1799 (a coefficient of variation of 3.36). The data for the awareness and preparedness are also quite uncertain so a variance of 0.02 times the mean is used, corresponding to a coefficient of variation ranging from 0.14 to 0.41. In 2006 only 21 interviews were conducted, while in 2002 and 2013 this was 300 and 117, respectively, so the uncertainty for 2006 is higher than for the other years (a variance of 0.03 times the mean is used, corresponding to a coefficient of variation ranging from 0.2 to 0.31). This results in the data points having distributions as shown in the right panel of Figure 3.

### 3.3 Prior Parameter Distributions

As explained in section 2.2, the posterior distribution of the parameters is calculated using the likelihood of the data and a prior distribution for the parameters. If information is available, it is possible to use an informative prior distribution; otherwise, a noninformative prior should be specified (Gelman et al., 2014). According to the literature, the half time of awareness, that is, the time after which awareness is halved, is 7 (International Commission for the Protection of the Rhine [ICPR], 2002) to 10 years (Bornschein & Pohl, 2014). Based on this information, an informative prior is constructed for *μ*_*A*_, the forgetfulness. We assume *μ*_*A*_ is normally distributed with a mean of 0.08 and a standard deviation of 0.015. Since the preparedness most likely has a slower decline than the awareness, we have chosen the prior for *μ*_*P*_ as normally distributed with a mean of 0.04 and a standard deviation of 0.03. In addition, *μ*_*A*_ is constrained to be higher than zero and *μ*_*P*_ to be in between zero and *μ*_*A*_; therefore, we truncated the normal distribution for both parameters. Since we do know the order of magnitude of the parameters values, we choose to use a truncated normal distribution instead of a lognormal distribution. No information is available for the other parameters except that they are constrained to be above 0. All other parameters have a truncated normally distributed prior with a mean of 0 and a standard deviation of 10, for *α*_*P*_ and *α*_*R*_, or 100, for *α*_*D*_ and *α*_*A*_ (since *α*_*P*_ and *α*_*R*_ cannot be too high, to avoid numerical problems).

The initial values for the awareness and the preparedness have been assigned an uninformative normal prior distribution with a mean of 0.5 and a standard deviation of 0.5, with *A*_0_ truncated between 0 and 1 and *P*_0_ truncated between 0 and *A*_0_, since the preparedness in 1798 must have been lower than the awareness (based on the definitions). The settlement density in 1780 has been calculated as 0.048, based on Gruner (2012); therefore, the *D*_0_ has been assigned a normally distributed prior with mean 0.048 and standard deviation 0.2 and is truncated between 0 and 1.

For the priors, we assume independence between the parameters and initial conditions, that is, we do not use multivariate distributions for the parameters, since the model is too complex for us to be able to say anything about their dependencies. This of course does not mean that the posterior distributions will also show independence.

## 4 Results

### 4.1 Time Series With Estimated Parameter Values

**Figure 4:**
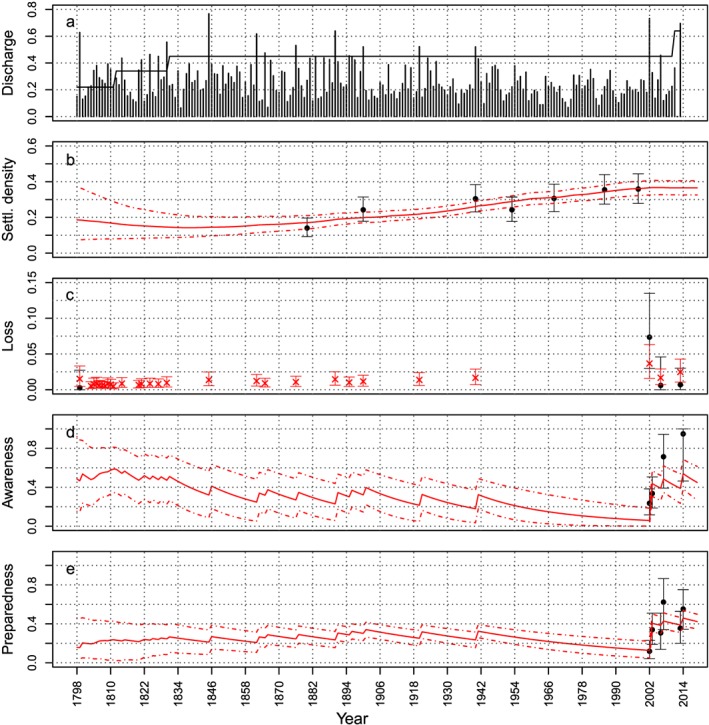
Discharge and protection level (input data) are plotted in (a). The evolution of the mean (solid line) and 95% credible bounds (dashed lines) of the fitted model variables over time are plotted in red (b–e). The empirical data (mean and 95% uncertainty bounds) that are used to estimate the parameter values are shown in black.

**Figure 5:**
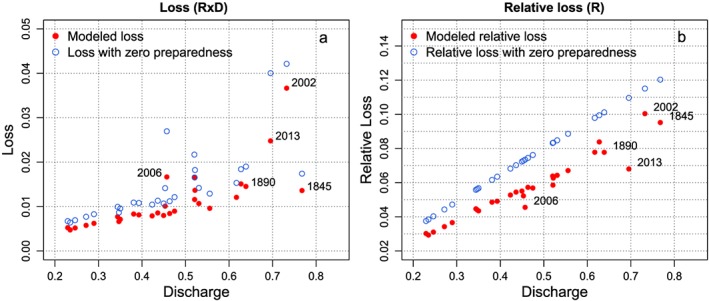
Estimated loss (red points) and loss if preparedness would have been zero (blue circles) versus normalized discharge (a). Estimated relative loss (red points) and relative loss if preparedness would have been zero (blue circles) versus normalized discharges (b). This figure shows the effects of only the preparedness, that is, only the relative loss (*R*), not taking into account the exposure at the time of the event (*D*).

**Figure 6:**
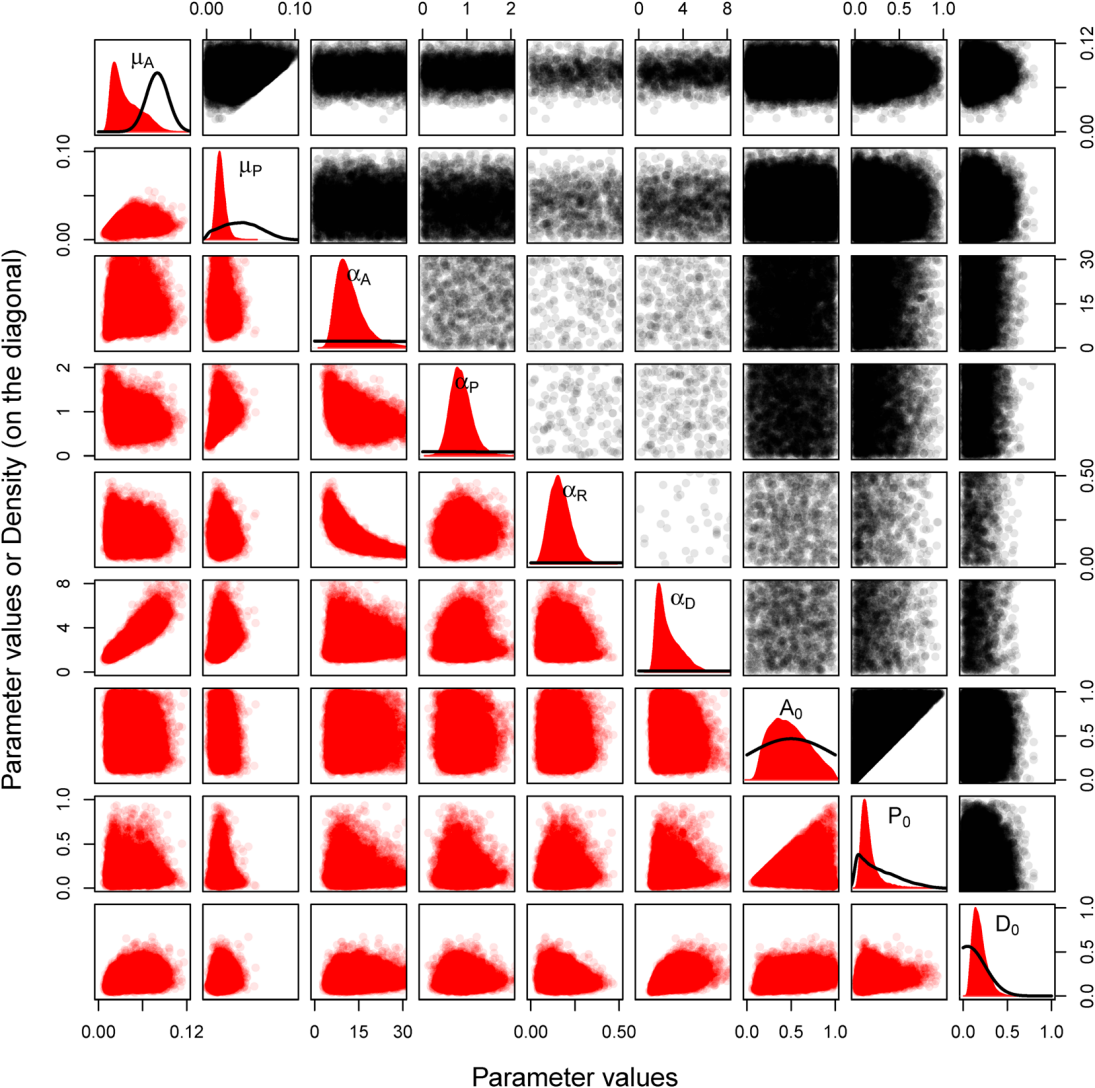
Model parameter and initial value prior (black) and posterior (red) distributions. The univariate density distributions are plotted in the panels on the diagonal, with the parameter values on the *x* axis and the density on the *y* axis. Above the diagonal the plots show samples from the bivariate prior distributions of the different combinations of parameters and below the diagonal the plots show samples from the bivariate posterior distributions of the different combinations of parameters. In these cases the axes represent parameter values.

The model described in section 2.1 is fitted to the data described in sections 3.1 and 3.2 (and shown in Figure 3) through the Bayesian MCMC method. The results of the fit are presented in Figures 4, 5, 6. Figure 4 shows the evolution of the variables over time given the estimated parameter distributions. The solid red lines give the mean estimated values, and the dashed lines show the 95% credible interval. Mean loss is shown as red crosses, and the whiskers provide the 95% credible bounds. The data used to estimate the parameter values are shown in black.

There is a slight increase in loss over time, due to an increasing settlement density. The model does seem to capture the effect of a raised awareness and the following increase in preparedness on the loss: due to an increase in awareness, the preparedness increases after 2002 and consequently the modeled loss in 2013 has a mean of 112 million € compared to a mean of 166 million € in 2002 (expressed in 2013 values for comparison).

Figure 5a shows the relationship between modeled loss (relative loss times the settlement density) and discharges when preparedness is included (red) and when preparedness is always zero (blue). The loss is not always high when the discharges are high. They vary due to changes in preparedness and also due to changes in settlement density as can be seen when comparing Figures 5a and 5b. Figure 5b shows the relationship between the relative loss and discharges. Depending on the level of preparedness, the (relative) loss is lower than it would have been if society had not been prepared. In 2002 preparedness was low and thus the difference between the modeled loss and the loss without preparedness is small. In 2013 (and in 2006), the preparedness has increased to 39% of the maximum possible value, meaning that on average the people in Dresden have implemented 39% of the measures they could have implemented, and therefore, relative loss is much lower than in 2002. Comparing Figure 5b to Figure 5a clearly indicates the influence of the settlement density or exposure on the size of the loss as well. The relative loss (Figure 5b) in 1845 is almost as high as in 2002 and the relative loss in 1890 was even higher than in 2013; however, because the settlement density was much lower, the actual loss (Figure 5a) is much lower in 1845 and 1890 than in 2002 and 2013, respectively.

Figure 4 shows that the preparedness at the start of the modeled period, although increasing, is not as high as it is at the end of the modeled period. This can be explained by the fact that the increase in preparedness depends on the increase in awareness, which in turn depends on the size of the loss. Since the settlement density influences the size of the loss, the loss in the first couple of years is lower than the loss at the end of the modeled period and as a consequence the preparedness is lower in that period, too.

According to the model, the awareness does not increase as much as the data suggest, even though the modeled awareness lies within the confidence bounds of the data, and society seems to forget less quickly than suggested by the literature, that is, *μ*_*A*_ is lower than expected. *μ*_*A*_ has a mean of 0.04, which is equivalent to a half time of 17 years and much longer than the 7 to 10 years that can be found in the literature (Bornschein & Pohl, 2014; ICPR, 2002). *μ*_*P*_ has a mean of 0.02, indicating that the preparedness has a half time of 34.5 years. Even though the forgetfulness of society seems to be lower than suggested in the literature, it is high enough for the awareness and preparedness to be almost zero in 2002, when the first flood in a period of more than 50 years occurred, meaning that on average almost 0% of the population in Dresden was aware of the risk and was prepared to deal with flooding. The awareness at the start of the modeling period is as high as at the end of the modeled period. This estimate seems to be reasonable since there were severe floods in 1784 and 1789 (Weikinn, 2000), just before the start of the modeling period, that may have raised awareness. The initial value of the awareness being so high, especially compared to the preparedness, results in the fact that during the period until 1845, on the long term, the awareness gradually decreases, while the preparedness gradually increases, because preparedness has a longer half time than the awareness. The preparedness has smaller credible bounds than the awareness in this period, which is a result of the fact that we have some qualitative data for the preparedness in the first couple of years.

The settlement density increases over time from 18% of the floodplain covered with households in 1798 to 37% covered in 2014 and does not seem to be influenced by the occurrence of floods that much. During the first 15 years, the credible bounds of the settlement density are quite big because we do not have any data available. During this period there may have been a decrease in the settlement density and thus a bigger effect of the awareness on settlement density. However, overall, the results indicate that there is almost no influence of households' awareness on the development of the settlement density over time.

### 4.2 Prior Versus Posterior Parameter Distributions

Figure 6 gives the prior (black) and posterior (red) distributions of the model parameters and the initial values. The marginal distributions are plotted in the panels on the diagonal, with the prior density distribution in black and the posterior density distribution in red. Samples from the bivariate prior and posterior distributions of the different combinations of parameters are plotted above and below it, respectively. The density of the points in the graphs above and below the diagonal is proportional to the prior and posterior probability of the parameters having a particular combination of values. Since we use flat uninformative priors for most of the parameters, the prior densities (in black above the diagonal) are very wide and in some cases most of the points fall outside the plotting area. The posterior densities (in red below the diagonal) are narrower, which indicates that the data provide information to update the parameter distributions.

As mentioned before, the forgetfulness *μ*_*A*_ is lower than expected and the posterior distribution of *μ*_*A*_ is shifted to the left compared to the prior distribution. Since the prior was constructed so that the value of *μ*_*P*_ should be lower than *μ*_*A*_, the posterior distribution of *μ*_*P*_ also lies to the left of the prior distribution. The other prior distributions were flat, weakly informative distributions, and they did not influence the posterior distributions much, as is apparent from the fact that we are able to identify the posterior distributions of these parameters with quite narrow credible bounds.

Since it was assumed that *P*_0_ should be below *A*_0_ and there is some information available about the preparedness in the beginning of the simulation period, the credible bounds of the preparedness are narrower than those of the awareness at the start of the modeled period and the posterior distribution of *P*_0_ is narrower than the posterior distribution of *A*_0_. The posterior distribution of *D*_0_ is narrower than the prior distribution, and *D*_0_ seems to be a bit higher than assumed a priori.

In general, there seems to be enough data for the posterior distributions not to be influenced too much by the prior distributions, even though the uncertainties in the data are assumed to be high. The prior distributions were assumed mutually independent. The posterior bivariate distributions show that for most parameters, this is indeed the case. However, there is a correlation between *μ*_*A*_ and *α*_*D*_ and between *α*_*A*_ and *α*_*R*_. When *μ*_*A*_ is small, that is, awareness declines less fast, the effect of the awareness on the growth of the settlement density, *α*_*D*_, is also small. This can be interpreted as the people in Dresden on average being either very forgetful (big *μ*_*A*_) and not very risk taking (big *α*_*D*_) or not so forgetful (small *μ*_*A*_) and very risk taking (small *α*_*D*_). The opposite is true for *α*_*A*_ and *α*_*R*_, when *α*_*R*_ is small and thus the preparedness has a smaller effect on the size of the loss; the increase in the awareness due to the loss, *α*_*A*_, is bigger. One could interpret this as the people of Dresden being very anxious (big *α*_*A*_) but the measures being not so effective in diminishing loss (small *α*_*R*_) or them being not so anxious (small α_A_) and the measures being very effective (big *α*_*R*_). Further information would be needed to better constrain these parameters.

### 4.3 Sensitivity Analysis

**Figure 7:**
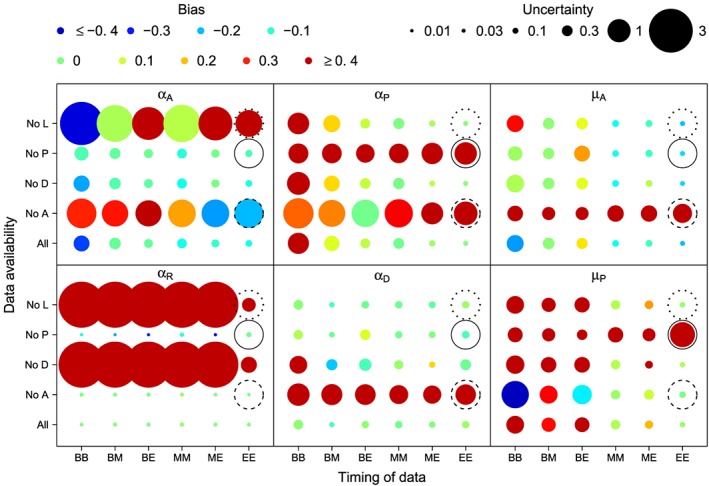
Bias and uncertainty of the parameter estimates for different combinations of data availability for the variables (all of them or leaving out data for awareness, settlement density, preparedness, or loss) and time of observation (B stands for at the beginning, M for in the middle, and E for at the end of the time series). Circled positions correspond to the time series plotted in Figure 8 (dotted circle), Figure 9 (solid circle), and Figure 10 (dashed circle).

The mean values of the posterior distribution of the model parameters (see Figure 6) are used to simulate synthetic time series for all variables. Then selected values of these variables are sampled as data, and the ability to correctly estimate the parameter values by using these data is investigated (as explained in section 2.3). Figure 7 shows the results of this sensitivity analysis. The six boxes present the sensitivity of estimation of the six model parameters. The colors of the points represent bias and their size variance (uncertainty) in parameter estimation. The grids contain each combination of available variables (whether all variables are observed or one of them is not) in each row and the timing of the observations in the columns. In general, the *α* parameters are most affected by leaving the data for one of the variables out of the inference, while the *μ* parameters are most affected by the timing of the observations. This is to be expected since the *α*s describe the relationship between the different variables, while the *μ*s determine the progress of a single variable over time.

As expected from the equations, the parameter that determines the effect of the loss on the awareness, *α*_*A*_, is most affected by the absence of data for awareness or loss. The absence of loss data in combination with the timing of the samples at the beginning of the modeled period causes the parameter to be underestimated, leading to the belief that the society is much less anxious than it is in truth, while taking samples at the end of the period causes an overestimation of the parameter, leading to the belief that the society is more anxious than it really is. The absence of awareness data has the opposite effect: a positive bias when samples are taken at the beginning and a negative bias when they are taken at the end of the modeled period.

The parameter relating the awareness and preparedness, *α*_*P*_, is most affected by not having awareness or preparedness data. The effect of not having awareness data changes with the timing of the samples, whereas the timing does not affect the effect of the absence of preparedness data.

The effects of the absence of data on the bias and uncertainty of the estimation of *α*_*R*_, which determines the relationship between preparedness and the relative loss, change depending on which type of data is missing. If data on loss or settlement density are missing, the bias is very large and positive and the uncertainty is high. If awareness data are absent, the bias is very small and the estimate has a low uncertainty, indicating that for estimating the loss parameters and dynamics right, the awareness data are not very important. The effect of missing preparedness data varies with the timing of the samples. In all cases the uncertainty is very small, but in some cases the bias is large and negative. This is particularly bad because the model suggests that something precise can be said about preparedness, which in reality is wrong.

The availability of settlement density does not seem to affect the estimation of *α*_*D*_, the effect of the awareness on the settlement density, very much. This is probably due to the fact that there is an external growth rate for the settlement density, making the actual settlement density data less important. The estimation of *α*_*D*_ is much more affected by the absence of awareness data. If the awareness data is not available, the bias in the estimation of *α*_*D*_ is very large.

The bias in the estimation of *μ*_*A*_ and *μ*_*P*_ (the decay rates of the awareness and preparedness) becomes very big and positive if there is an absence of awareness or preparedness data, respectively. In this case the parameter values are overestimated. The availability of other variables does not affect the bias or uncertainty that much. Instead, the timing of the samples has a much bigger influence on the estimation of *μ*_*A*_ and *μ*_*P*_. This also follows from the equations because these parameters describe the changes in time of a certain variable and not the relationship between two variables. For the estimation of both parameters two data points at the beginning of the modeled period give the highest uncertainties in the estimation of the parameters. The bias in the estimation of *μ*_*P*_ is also largest with this timing of the samples and smallest if samples are taken at the end of the period. In this case, however, the estimate for *μ*_*A*_ has the highest bias, and a small uncertainty, meaning that after the inference we think we know the parameter with high precision, but in fact we are underestimating its value. Biases are small if a sample in the middle is combined with a sample at the beginning or at the end.

Overall, having two data points at the end of the period is best, since both bias and uncertainty are low or relatively low. This was to be expected since the present state (i.e., at the time when data are available) holds information about the past and how the situation ended up as it is (if the system does not forget very quickly), but not about the future. The parameters *α*_*A*_ and *μ*_*A*_ are an exception to this, since they are both overestimated when samples are taken at the end of the period. Thus, after the estimation we would think that the households are less anxious (increasing the awareness not so much after an event) and also forget less fast, while in fact they are more anxious and they forget faster.

**Figure 8:**
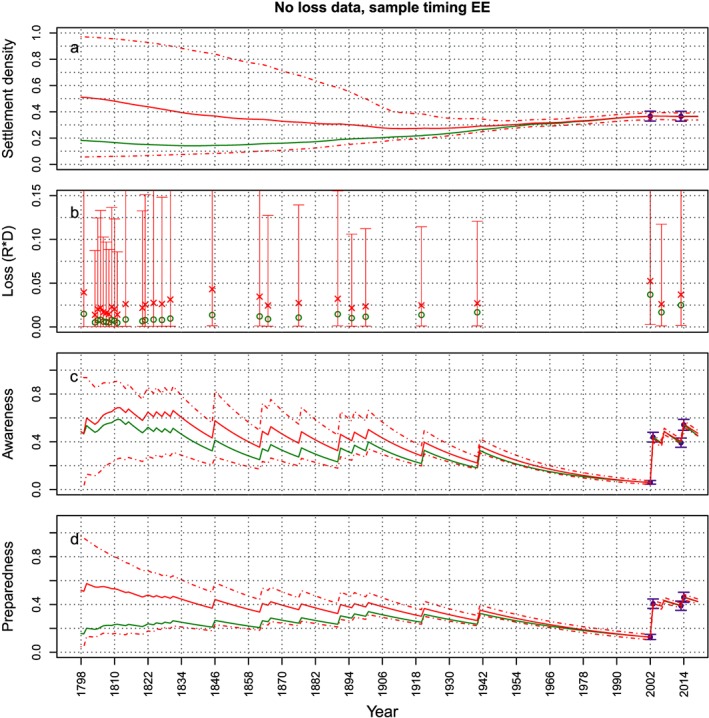
The synthetic time series from which data are sampled are plotted in green. Data points that are sampled from the synthetic time series and used to estimate the parameter values are plotted in purple (mean and 95% uncertainty bounds). The evolution of the mean (solid line) and 95% credible bounds (dashed lines) of the fitted model variables over time are plotted in red. In this case both samples are taken at the end of the modeled period and loss data is missing (corresponding to the dotted circle in Figure 7). An absence of loss data mostly affects the estimation of the loss. The other variables are estimated quite well.

**Figure 9:**
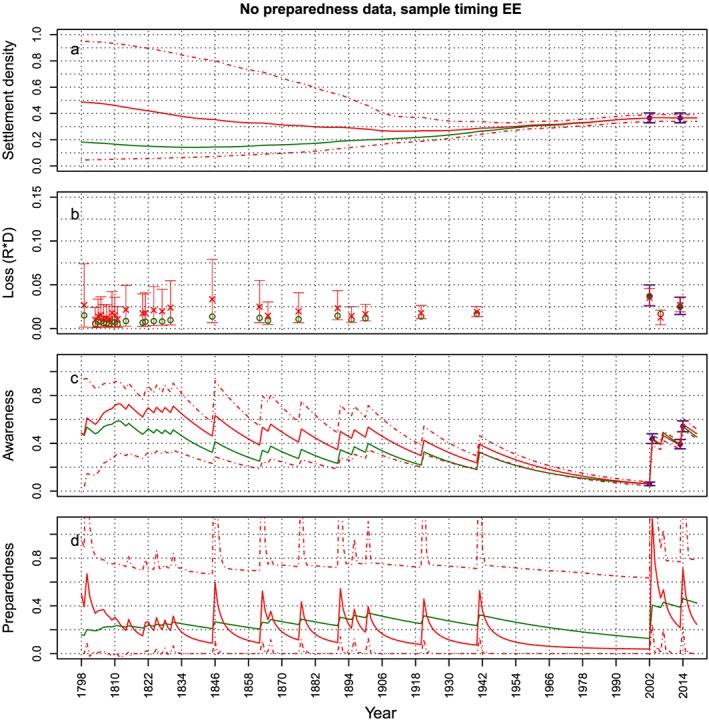
Same as Figure 8 but in this case preparedness data are missing (corresponding to the solid circle in Figure 7). An absence of preparedness data affects the estimation of the preparedness. The loss is overestimated and has bigger credible bounds than in the case where preparedness data is available, other than that the other variables are estimated quite well.

**Figure 10:**
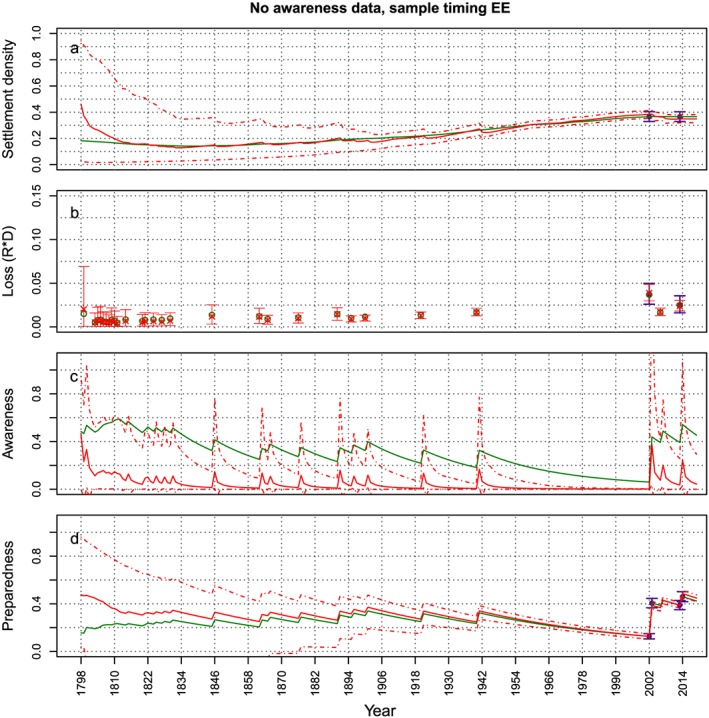
Same as Figure 8 but in this case awareness data are missing (corresponding to the dashed circle in Figure 7). An absence of awareness data mostly affects the estimation of the awareness and settlement density. The other variables are estimated quite well.

In order to better present some results of the sensitivity analysis, Figures 8, 9, 10 show the effect of not having data for the awareness, preparedness, or loss, respectively (i.e., the cases *No A – EE*, *No P – EE*, and *No L – EE*), on the estimation of the variables, when samples are taken at the end of the modeled period. For all three cases, the effect of not having data on the estimation of the model variables is highest for the variable for which data are absent, which is to be expected. In case loss data is missing, the estimation of the other variables does not seem to be affected that much (Figures 8c and 8d). The absence of preparedness causes a small overestimation of the loss with large credible bounds (Figure 9b). The figures do show that at the start of the period, the estimated mean of most variables is quite far off from the synthetic time series, but this seems to be due to the incorrect estimation of the initial values and not because the behavior of the system is not estimated correctly. The dynamics of the behavior of the estimated variables over time is similar to the synthetic data time series. In case awareness data is missing, however, the dynamics (i.e., the oscillations) of the estimated settlement density are very different from the dynamics of the *real* settlement density (Figure 10a): the estimated time series goes up and down, whereas the real time series are smooth. This occurs because the estimate of *α*_*D*_ has a large positive bias, which means that we think the awareness has a much bigger influence on the settlement density than it has in reality. The fact that missing awareness data has such a big influence on the inference may be because it is such an important variable in the model that is proposed in this study. From equations (1a)–(1e) one can see that it influences both the preparedness and the settlement density, so if awareness data are missing it becomes very difficult to estimate the model dynamics correctly.

**Figure 11:**
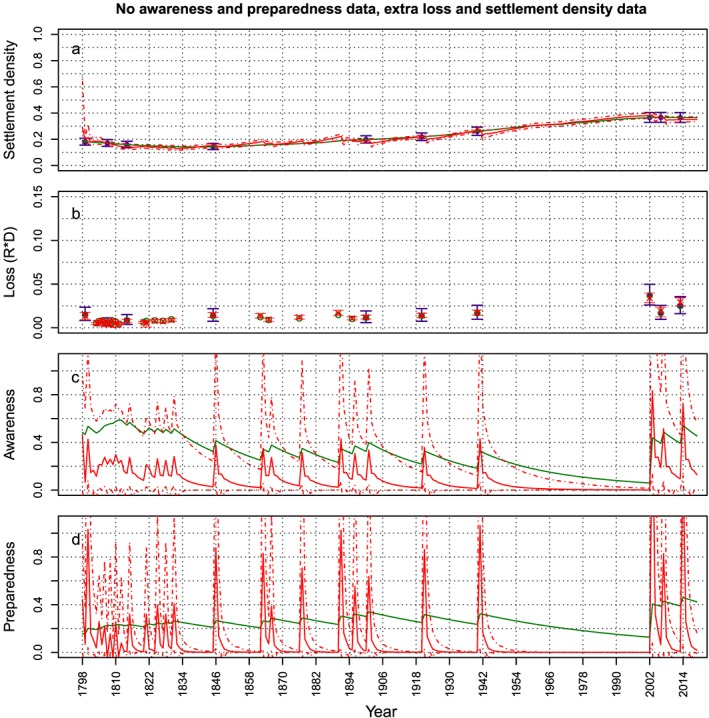
Same as Figure 8 but in this case both awareness and preparedness data are missing and 10 data points for loss and settlements density are used.

To investigate whether we can make up for an absence of awareness data by using more loss and settlement density data, we redo the analysis using 10 data points for the settlement density and loss (see Figure 11) and no data for the awareness or preparedness. We also exclude the preparedness because both kinds of data are collected with the use of surveys; therefore, if awareness data are not available, preparedness data will not be available either. Figure 11 shows that this does not solve the problem, as both awareness and preparedness are not estimated correctly and the estimated time series of the settlement density still show oscillations indicating that the parameter α_D_ is still overestimated.

## 5 Discussion and Conclusion

**Figure 12:**
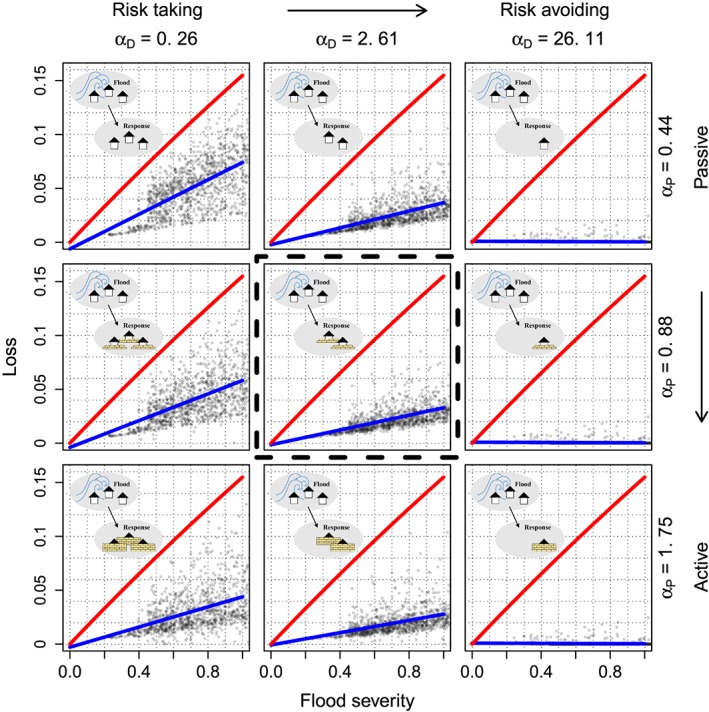
The panels show the effect of different behavioral attitudes after experiencing a flood on the expected flood loss as a consequence of floods with a flood magnitude from zero to one. The red line gives the loss if preparedness is zero, and the settlement density is one (a completely settled floodplain). The blue line gives the loss as modeled with the parameter values shown on the axes. From left to right the panels show a change in *α*_*D*_, the risk taking attitude, from risk taking to risk avoiding. From top to bottom the panels show a change in *α*_*P*_, the activeness, from passive to active. The human‐flood system of Dresden is located in the panel indicated with a black dashed square.

This paper demonstrates that studying specific case studies and using empirical data to estimate the parameters of sociohydrological models provides us with many insights into the dynamic interactions between humans and floods. In the case of Dresden, the inference shows that increasing awareness leads society to take precautionary measures to reduce loss rather than decrease exposure. It also allows for investigating *hidden* factors, like the forgetfulness of a society. In the case of Dresden, the inference indicates that after about 17 years, society has lost half of the awareness accumulated after an event, which is longer than what literature suggests (a half time of 7 to 10 years, Bornschein & Pohl, 2014; ICPR, 2002). Also, the model allows for the inference of the development of variables at time points where no data are available. For example, in the case of Dresden, losses after the 1845 and 1890 floods have been quantified to be similar to the one in 2006, because even if exposure was lower in the nineteenth century than in 2006, the preparedness was lower too. The modeling procedure presented in this paper therefore produces quantitative hypotheses that may be checked by retrieving other data. Figure 12 shows the effect of risk taking attitude (the parameter *α*_*D*_) and activeness (the parameter *α*_*P*_) on the behavior of a society, as represented in Figure 1. The panels are filled with values of loss and related flood magnitudes generated by the model. They show the decrease in expected loss (the difference between the blue line and the red line) as a result of decreases in settlement density and increases in preparedness. The size of the decrease in loss depends on the values of *α*_*D*_ and *α*_*P*_. For the case study of Dresden, the parameter values are those of the panel in the middle (indicated by a black dashed square). Applying the same model and parameter estimation procedure to other case studies will enable a comparison between their behavior and its consequences.

We have investigated the usefulness of data availability through a sensitivity analysis. We show that it is most useful to have data at the end of the modeled period. In that case, the estimates of the parameters have the smallest bias, with the exception of the estimates for the parameters determining the dynamics of the awareness. Since for the case study of Dresden we mostly have data at the end of the time period, this may indicate that the forgetfulness of Dresden (equivalent to a half time of 17 years) could have been underestimated and that the real value is in fact closer to the value that was found in the literature (a half time of 7 to 10 years and thus a bigger forgetfulness *μ*_*A*_ (Bornschein & Pohl, 2014; ICPR, 2002). In general, the fact that having data at the end of the modeling period is most useful is good news, since for most sociohydrological systems, this is the time when data is usually available. However, it should be noted that prediction of future dynamics is difficult and that it should be done with caution. The prediction should rather be a *mapping of possibility space* (Srinivasan et al., 2017) taking into account all the different possible futures. The estimation of the parameter values as proposed in this paper does allow for the determination of the state of the system at present, which provides a better starting point for mapping the possibility space.

Which type of data has the most value for the estimation of the parameters depends on what is of most importance to the model user. The sensitivity analysis shows that data about loss and settlement density are least important, unless the user is most interested in modeling the loss with a higher degree of accuracy. In contrast, data about awareness are very important for the estimation of all parameters. If awareness data are absent, most of the parameter estimations are biased and the modeled dynamics of the variables over time is wrong. Preparedness data are also important, especially when the modeler is interested in estimating the preparedness and/or the loss more accurately. However, if preparedness data are missing the behavior of the variables over time is still modeled correctly, even though the mean estimated preparedness is biased. Awareness data are not only the most important data for estimating the parameter values correctly, it is also the most difficult and most expensive data to obtain (together with preparedness data), because the data is gathered through surveys, which are rather time consuming and expensive. Unfortunately, the analysis shows that it is not possible to correct for missing awareness and preparedness data by using more data points of the more easily obtainable loss and/or settlement density. In this specific model structure the awareness is a very important variable and has a big influence; therefore, awareness data are so important. In cases with model structures where awareness plays a less important role, not having data on awareness may have less influence on the estimation of the parameter values. However, in general, one could say that awareness is a very important factor in human‐flood interactions and it will most probably be included in most models, therefore, easier, less costly methods to collect awareness and preparedness data than using surveys should be sought, for example, using social media (e.g., Vieweg et al., 2010).

As this paper shows, even when uncertainty in the data is high, it is feasible to estimate the parameters of sociohydrological models. We have used Bayesian inference to accommodate data uncertainty, which results in estimated uncertainties in the estimated model parameters. However, as explained in section 1.2, measuring social processes is difficult and the values of the social variables for the system of Dresden are approximated based on stated answers rather than the observed behavior, which may be very different. This means that the data used for the parameter estimation may be biased or misinterpreted and one should keep this in mind when model results are used for further analysis or policy making.

While uncertainty in the data is accounted for, the Bayesian inference does not account for the fact that the model could miss some significant processes. Other models could give a different hypothesis of the most important variables in the system and their interactions, which could be tested and compared to the model proposed here. For example, the model proposed here is a lumped model, that is, the variables represent an average value for society. The literature on empirical data studies indicates that households can have different reasons for implementing precautionary measures or not (Bubeck et al., 2012, 2013; Miceli et al., 2008; Raaijmakers et al., 2008; Scolobig et al., 2012; Wachinger et al., 2013), but in this case, these are all represented by a single parameter. While this model does not tell us anything about individual household choices, it does give an indication about the average uptake of precautionary measures and the changes over time. In future work, the model could be extended to account for the different coping mechanisms more explicitly, to model households separately or to include, for example, real estate prices and insurance incentives as variables in the model. Also, we assume that the model parameters are constant values in time, while they may evolve (slower than the other variables) due to cultural changes. To test different hypotheses, different models could be developed and their performance compared with Bayesian inference (Gelman et al., 2014).

The authors believe that the method presented here is an important step in the field of sociohydrological research. For the case of Dresden, the model structure, and thus the hypothesis about the processes and feedback mechanisms, seems to be consistent with the data. The same model could be fit to other case studies that have similar feedback mechanisms but may have different characteristics, for example, a higher or lower forgetfulness. This would allow for performing comparative sociohydrology among case studies, which have the same possible development trajectories but may follow a different particular trajectory. Comparing and contrasting different systems is the way forward to advancing the general understanding about the sociohydrology of human‐water systems (Sivapalan et al., 2012).

## Supporting information



Supporting Information S1
