# Supplementary material for: The Value of Empirical Data for Estimating the Parameters of a Sociohydrological Flood Risk Model
Source: Water Resour Res. 2019 Feb 15;55(2):1312–36. doi: 10.1029/2018WR024128 (PMC6472491; doi:10.1029/2018WR024128)
Supplement: Supplementary file 1 — Supporting Information S1 [file WRCR-55-1312-s001.docx]

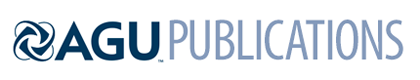


*Water Resources Research*

Supporting Information for

**The value of empirical data for estimating the parameters of a socio-hydrological flood risk model**

M. H. Barendrecht1, A. Viglione1, H. Kreibich2, B. Merz2,3, S. Vorogushyn2, G. Blöschl1

1Centre for Water Resource Systems, Vienna University of Technology, Vienna, A-1040, Austria.

2GFZ German Research Centre for Geosciences, Section Hydrology, Telegrafenberg, Potsdam, 14473, Germany.

3University of Potsdam, Institute of Earth and Environmental Sciences, Potsdam, 14476, Germany.

**Contents of this file**

Text S1

Tables S1

**Introduction**

The supporting information includes a description of the implementation of the Bayesian Inference and a table with the values of the data as used for the inference. Descriptions of data and data processing are included in the main manuscript.

Text S1. Appendix A Implementation Bayesian Inference

Equations A1.1 to A1.6 describe the system of differential equations from equation 1 in discretized form using forward Euler. We use a time step of 1 year.

$$\begin{aligned} L_{t}=R_{t}D_{t} \#\left( A1.1 \right) \end{aligned}$$

$$\begin{aligned} R_{t}=\left\{ \begin{aligned} R_{max}-\beta_{R}\exp\left( -\alpha_{R}\left( P_{max}-P_{t} \right)\frac{W_{t}}{W_{max}} \right), &W_{t}>H_{t} \\ 0, &W_{t}\leq H_{t} \end{aligned} \right.\#\left( A1.2 \right) \end{aligned}$$

$$\begin{aligned} D_{t+1}=D_{t}+\left( U_{t}\left( 1-\alpha_{D} \right)D_{t}\left( 1-\frac{D_{t}}{D_{max}} \right) \right)\Delta t\#\left( A1.3 \right) \end{aligned}$$

$$\begin{aligned} A_{t+1}=A_{t}+\left( \alpha_{A}L_{t}\left( 1-\frac{A_{t}}{A_{max}} \right)-\mu_{A}A_{t} \right)\Delta t\#\left( A1.4 \right) \end{aligned}$$

$$\begin{aligned} P_{t+1}=P_{t}+\left( \alpha_{P}\frac{\left( A_{t+1}-A_{t} \right)}{\Delta t}\left( 1-\frac{P_{t}}{P_{max}} \right)S\left( R_{t} \right)-\mu_{P}P_{t} \right)\Delta t\#\left( A1.5 \right) \end{aligned}$$

$$\begin{aligned} S\left( R_{t} \right)=\tanh\left( 1000R_{t} \right)\#\left( A1.6 \right) \end{aligned}$$

The parameters µ_A_, µ_P_, α_A_, α_P_, α_R_ and α_D­_ are estimated using Bayesian Inference. The likelihood function describing the likelihood of the data given the parameter values is given by eq. A2:

$$\begin{aligned} \mathcal{l}\left( D | \theta\right)=\left( \prod_{i=1}^{N} f_{\beta}\left( D_{i};M_{i}\kappa_{i},\left( 1-M_{i} \right)\kappa_{i} \right) \right)f_{N}\left( \frac{P_{\left( t=1846 \right)}}{P_{\left( t=1798 \right)}};2,0.5 \right)\#\left( A2 \right) \end{aligned}$$

*θ* represent the parameters to be estimated: $\theta=\left( \alpha_{A}{,\alpha}_{P},\alpha_{D},\alpha_{R},\mu_{A},\mu_{P} \right)$. *D* are the data points for D, A, P and L and *M* the corresponding modelled values, with *κ* representing the uncertainty in the data point. We assume the data are distributed according to a beta distribution $f_{\beta}\left( y;\mu\nu,\left( 1-\mu\right)\nu\right)$, with mean µ and sample size ν. The sample size can be expressed as $\frac{\mu\left( 1-\mu\right)}{variance}-1$, and thus represents the uncertainty in the data (as explained in section 3.2). The second term to the right in eq. A2 represents the knowledge that P increased in the period between 1798 and 1846. We assume that the ratio of P at these two points in time is distributed according to a normal distribution $f_{N}\left( y;\mu,\sigma\right)$, with a mean of 2 and a standard deviation of 0.5. The inference was performed using Stan (Carpenter et al., 2017), with prior distributions for the parameters as described in section 3.3.

| **Variable** | **Time** | **Value** | **Data source** |
| --- | --- | --- | --- |
| Settlement density D | 1880, 1900, 1940, 1953, 1968, 1986, 1998 | 0.141, 0.243, 0.305, 0.243, 0.307, 0.355, 0.359 | Gruner, 2012 |
| Urbanisation rate U | 1798 - 1879, 1880 - 1889,  1890 - 1899, 1900 - 1909, 1910 - 1919, 1920 - 1929,  1930 - 1939, 1940 - 1949,  1950 - 1959, 1960 - 1969,  1970 - 1974, 1975 - 1979, 1980 - 1984, 1985 - 1989,  1990 - 1994, 1995 - 1999,  2000 - 2004, 2005 - 2009,  2010 - 2014 | 0.024, 0.032, 0.031, 0.036, 0.013, 0.029, 0.025, 0.031, 0.020, 0.006, 0.014, 0.007, 0.012, 0.011, 0.008, 0.004, 0.006, 0.011, 0.006 | Paprotny, 2017 |
| Awareness A | 2002, 2003, 2007, 2014 | 0.237, 0.337, 0.714, 0.949 | Kreibich et al., 2005; Kreibich and Thieken, 2009; Thieken et al., 2016 |
| Preparedness P | 2002, 2003, 2006, 2007, 2013, 2014 | 0.119, 0.340, 0.307, 0.623, 0.356, 0.553 | Kreibich et al., 2005; Kreibich and Thieken, 2009; Thieken et al., 2016 |
| Losses L | 1799, 2002, 2006, 2013 | 0.003, 0.074, 0.006, 0.007 | Korndorfer, 2006; Kreibich et al., 2011; LfULG, 2015;  Kleist et al. 2006; IKSE, 2007;  Kreibich et al., 2005; Weikinn, 2000; Poliwoda, 2007; Deutsche Bundesback |
| Floods W | 1798 - 2013 | 0.151, 0.627, 0.130, 0.153, 0.220, 0.229, 0.344, 0.381, 0.290, 0.272, 0.246, 0.393, 0.350, 0.235, 0.287, 0.237, 0.436, 0.272, 0.154, 0.181, 0.125, 0.109, 0.347, 0.423, 0.114, 0.163, 0.464, 0.181, 0.147, 0.450, 0.274, 0.294, 0.556, 0.229, 0.239, 0.142, 0.341, 0.064, 0.203, 0.320, 0.393, 0.254, 0.268, 0.341, 0.197, 0.206, 0.268, 0.768, 0.390, 0.319, 0.315, 0.166, 0.379, 0.241, 0.323, 0.297, 0.254, 0.375, 0.332, 0.142, 0.257, 0.155, 0.387, 0.234, 0.617, 0.113, 0.123, 0.475, 0.069, 0.419, 0.304, 0.193, 0.180, 0.338, 0.329, 0.111, 0.158, 0.216, 0.531, 0.359, 0.229, 0.142, 0.270, 0.435, 0.117, 0.448, 0.134, 0.145, 0.438, 0.180, 0.426, 0.252, 0.639, 0.409, 0.249, 0.220, 0.237, 0.453, 0.449, 0.423, 0.142, 0.336, 0.521, 0.194, 0.122, 0.202, 0.150, 0.136, 0.253, 0.235, 0.162, 0.387, 0.167, 0.188, 0.132, 0.181, 0.246, 0.383, 0.206, 0.365, 0.139, 0.185, 0.522, 0.144, 0.209, 0.437, 0.340, 0.225, 0.410, 0.240, 0.219, 0.197, 0.157, 0.222, 0.280, 0.174, 0.093, 0.201, 0.173, 0.196, 0.235, 0.205, 0.520, 0.431, 0.259, 0.096, 0.321, 0.248, 0.371, 0.382, 0.351, 0.200, 0.116, 0.161, 0.176, 0.222, 0.379, 0.265, 0.299, 0.204, 0.332, 0.118, 0.168, 0.196, 0.184, 0.088, 0.089, 0.299, 0.224, 0.254, 0.218, 0.180, 0.226, 0.133, 0.107, 0.068, 0.129, 0.309, 0.233, 0.306, 0.206, 0.228, 0.272, 0.352, 0.283, 0.134, 0.084, 0.160, 0.223, 0.276, 0.343, 0.192, 0.142, 0.104, 0.171, 0.167, 0.267, 0.219, 0.192, 0.201, 0.181, 0.274, 0.267, 0.180, 0.732, 0.328, 0.137, 0.274, 0.457, 0.119, 0.163, 0.189, 0.225, 0.363, 0.000, 0.695 | BMBF-Projekt; Umweltamt Dresden, personal communication, July 19, 2017 |
| Protection level H | 1798 - 1811, 1812 - 1830, 1831 - 2010, 2011 - 2013 | 0.220, 0.340, 0.450, 0.639 | Pohl, 2004; Weikinn, 2000; Weikinn & Börngen, 2002; Landestalsperrenverwaltung des Freistaates Sachsen, 2013; Umweltamt Dresden, personal communication, July 19, 2017 |

Table S1. Data used for the inference. Data and calculations are described in the main manuscript.
